# Supplementary material for: Intermediate filaments associate with aggresome-like structures in proteostressed C. elegans neurons and influence large vesicle extrusions as exophers
Source: Nat Commun. 2023 Jul 24;14:4450. doi: 10.1038/s41467-023-39700-1 (PMC10366101; doi:10.1038/s41467-023-39700-1)
Supplement: Supplementary file 3 — Description of Additional Supplementary files [file 41467_2023_39700_MOESM3_ESM.docx]

**Intermediate Filaments Associate with Aggresome-like Structures in Proteostressed *C. elegans* Neurons and Influence Large Vesicle Extrusions as Exophers**

**Supplementary Data**

**Supplementary Videos. Demonstration of the distinctive structural differences between membrane layers and intermediate filament types.**  P*_mec-4_*mCherry animals were staged as adult day 2 with an exopher bud phenotype.

**Video 1:** Video highlighting whorl membrane geometry. Movie 1 shows a focus through series in which individual membranes continue through the full depth of the series without dropping out (unlike members of a filament bundle). Membranes often lie in closely packed layers but may bend sharply to double back on themselves. Scale bar is 50 nm.

**Video 2:** Video highlighting intermediate filaments associated with the circular organelle. Movie 2 shows filaments in a focus-through series demonstrates that (unlike membranes) these filaments do not continue through the full depth of the series, but individually disappear (or appear) at different depths within the series. Some longer filaments run for a distance around the organelle, bending gradually, but these filaments are loosely associated and rarely touch one another. Filaments deeper inside the organelle tend to be short, and run at odd angles to one another, rarely pairing. Scale bar is 50 nm.

**Supplementary Fig. 1. IFD-1 compartments are evident and enlarge under neuronal stress conditions. a-f** Error bars indicate SEM. **a** Number of IFD-1 inclusions per ALM neuron with increasing age. Strain is expressing *bzIs166*[P*_mec-4_*mCherry]; *bzSi3*[P*_mec-7_*GFP::IFD-1]. Life-stage points assayed: L4, Ad1, Ad2, Ad3, Ad4, Ad6, Ad10, Ad11, Ad12, Ad14. N = 25, 19, 34, 28, 30, 25, 9, 13, 12 for lifestages respectively. One-way ANOVA, Dunnett’s post-test. Ad1 **P = 0.0096, Ad2 * P = 0.038, Ad3 ***P = 0.002. **b** IFD-1-positive inclusions increase in size with age. GFP::IFD-1 diameter in ALM neurons in µm. Timepoints and N as in **a**. Error bars are SEM. **c** IFD-1-positive puncta increase in size under enhanced proteostress induced by mCherry expression. GFP::IFD-1 diameter in ALM neurons on Ad2, in µm. Strains are *bzSi3*[P*_mec-7_*GFP::IFD-1] and *bzIs166*[P*_mec-4_*mCherry]; *bzSi3*[P*_mec-7_*GFP::IFD-1], N = 69 and 51 puncta respectively. **P = 0.0027, 3 trials, two-tailed t-test. **d** IFD-1-positive puncta number per cell increases in the *epg-9/*ATG101 mutant*.* Strains are *epg-9(bp320)*III; *bzIs166*[P*_mec-4_*mCherry]; *bzSi3*[P*_mec-7_*GFP::IFD-1] and *bzIs166*[P*_mec-4_*mCherry]; *bzSi3*[P*_mec-7_*GFP::IFD-1]. N = 39 and 41 ALM respectively. P = 0.07, 3 trials, two-tailed t-test. **e** Maximum IFD-1-positive puncta increase in size in *epg-9/*ATG101 mutant*.*  Strains are *epg-9(bp320)III*; *bzIs166*[P*_mec-4_*mCherry]; *bzSi3*[P*_mec-7_*GFP::IFD-1] and *bzIs166*[P*_mec-4_*mCherry]; *bzSi3*[P*_mec-7_*GFP::IFD-1]. N = 38 and 42 puncta respectively. Maximum GFP::IFD-1 punctae diameter per ALM, in µm. *P = 0.028, 3 trials, two-tailed t-test. **f** IFD-1-positive puncta increase in size under enhanced proteostress induced by proteasome and autophagy inhibitors. We treated a strain expressing *bzIs166*[P*_mec-4_*mCherry]; *bzSi3*[P*_mec-7_*GFP::IFD-1] with either DMSO or both 10 mM proteasome inhibitor MG132 and 10 mM autophagy inhibitor Spautin-1, proteostressors that are known to enhance exopher production, from L4 to Ad3, and measured GFP::IFD-1 inclusion size at Ad3. N = 85 and 50 puncta for the respective condition. GFP::IFD-1 diameter in µm. *P = 0.014, 3 trials, two-tailed t-test. **g-j.** Adult ALM soma outlined with continuous line, nucleus outlined with dotted-line. Scale bar = 2 µm. Images representative of N > 10 ALM. **g** *bzSi34*[P*_mec-7_*mSc::IFD-1] **h** *bzSi3*[P*_mec-7_*GFP::IFD-1] **i**  *bzSi37*[P*_mec-7_*mNG::IFD-2]; *bzIs166*[P*_mec-4_*mCherry] **j** *bzEx253*[P*_mec-7_*RFP::IFD-2]; *zdIs5*[P*_mec-4_*GFP].

**Supplementary Fig. 2. Ultrastructural analysis reveals the presence of intermediate filament assemblies in distinctive perinuclear structures in proteo-stressed ALM neurons.**Touch neurons in P*_mec-4_*mCherry animals at Ad2 show early exopher buds still within the ALM soma (12 ALMs examined in TEM). **a** Soma in transverse section displays a distinctive circular structure (white arrow) close to the nucleus (Nuc) and a small mitochondrion (M). Electron lucent contents include short filaments ~ 10 nm in width at random angles within the organelle, with discontinuous filaments of similar width coating the periphery as a single layer. A bundle of microtubules lies inside the soma, just beneath the hypodermis and cuticle. Short black arrows mark the soma plasma membrane. Scale bar 500 nm. **b** Higher magnification of the circular organelle from panel A again shows the filaments (white arrows). Red arrow indicates a longer filament at the outer edge of the organelle. Note the thinness of IFs compared to the membrane bilayer surrounding a nearby mitochondrion. See also Supplementary Videos 1 and 2 that compare IFs to plasma membrane appearance. Scale bar 200 nm. **c** Another similar organelle in ALM soma of a different animal where intermediate filaments (red arrows) assemble into a loose bundle that curves as it extends along the organelle’s periphery. A few short filaments lie deeper inside the organelle. Scale bar is 200 nm. **d** Electron tomogram in another ALM shows curving individual filaments bordering a circular organelle, with a denser, tighter accumulation of filaments (between red arrows) at the edge of the organelle. A bundle of microtubules (blue arrowheads) lies just outside the organelle. Scattered dark ribosomes are seen within the structure, plus granular matrix material of moderate electron density that likely represents proteinaceous aggregates. Scale bar is 200 nm. Although successive sequencing is not possible for individual samples, we speculate that the juxtanuclear structures mature by increasing and expanding the proteinaceous center and adding more intermediate filaments to the periphery. Overall, limited low resolution EM data exist on mammalian aggresome-focused EM^1-8,9^. Although the initial description describing IF cages was based on fluorescent microscopy^3^, available EM data suggest at best loose and somewhat disorganized IF networks in aggresome structure, similar to what we report here.

**Supplementary Fig. 3. Disruption of *hda-6*, *sqst-1,* and *ftt-2/*14-3-3 can modulate IFD compartment size, but no single disruption eliminates the aggresome-like compartment.** Statistics are two-tailed t-test, error bars are SEM.  **a** Maximum IFD-1 puncta size decreases in *sqst-1*(*ok2892*)*.* *sqst-1* encodes the ortholog of human SQSM1, an aggregate adapter protein implicated in mammalian aggresome formation and autophagy. Strains are *bzIs166*[P*_mec-4_*mCherry]; *bzSi3*[P*_mec-7_*GFP::IFD-1] and *bzIs166*[P*_mec-4_*mCherry]; *sqst-1ok2892*); *bzSi3*[P*_mec-7_*GFP::IFD-1]; N = 29 and 35 puncta respectively, diameter of the largest IFD-1 puncta in ALM Ad2 in µm; P = 0.0075, 3 trials. **b** IFD-1 puncta number per ALM increases in *sqst-1*(*ok2892*)*.* Strains are WT or *(sqst-1(Δ))* as in **a***,* N = 28 and 34 ALMs, respectively. Number of IFD-1 puncta per Ad2 ALM indicated, P = 0.014, 3 trials. **c** Maximum IFD-1-positive puncta size decreases in *ftt-2(n4426).* Diameter, in µm*,* of the largest Ad1 IFD-1-positive puncta per ALM neuron decreases in the *ftt-2* likely null background, in a strain expressing *bzIs166*[P*_mec-4_* mCherry]; *bzSi3*[P*_mec-7_* GFP::IFD-1]; N = 114 and 112 puncta in control and mutant, respectively; P = 0.0001, 3 trials. We measured on Ad1 to reduce animal-bagging and egg retention consequences. **d** IFD-1-positive puncta number per ALM neuron remains the same in control and *ftt-2(n4426).* Number of IFD-1-positive inclusions in Ad1 ALM somas does not change in mutant *ftt-2(n4426)* versus WT in strains expressing *bzIs166*[P*_mec-4_* mCherry]; *bzSi3*[P*_mec-7_* GFP::IFD-1]. N = 42 and 40 Ad1 ALM respectively; NS, P = 0.87, 3 trials. We measured on Ad1 to reduce animal-bagging consequences. **e** HDA-6 is an ortholog of human HDAC6, a cytoplasmic deacetylase with conserved function as an adapter, microtubule modulator, and autophagy regulator. *hda-6(ok3203)* does not affect average IFD-1 puncta size in *bzIs166*[P*_mec-4_*mCherry] background. Strains are *bzIs166*[P*_mec-4_*mCherry]; *hda-6(ok3203),* *bzSi3*[P*_mec-7_*GFP::IFD-1] and *bzIs166*[P*_mec-4_*mCherry]; *bzSi3*[P*_mec-7_*GFP::IFD-1]. N = 81 and 72 puncta respectively. Diameter of IFD-1 puncta measured in ALM on Ad2 in µm, 4 trials. **f** *hda-6(ok3203)* does not significantly affect IFD-1 puncta number per cell in the *bzIs166*[P*_mec-4_*mCherry] background. Strains *bzIs166*[P*_mec-4_*mCherry]; *hda-6(ok3203); bzSi3*[P*_mec-7_*GFP::IFD-1] and *bzIs166*[P*_mec-4_*mCherry]; *bzSi3*[P*_mec-7_*GFP::IFD-1], N = 48 and 45 ALM, respectively. Number of IFD-1 puncta per ALM Ad2, 4 trials.

**Supplementary Fig. 4. Disease-associated human Htt-polyglutamine expansion protein dynamically colocalizes with IFD-1 in touch neurons. a** HttQ128::CFP and mSc::IFD-1 compaction over time. Images are selected from a 5-day time-course of an ALM soma; neuron expressing HttQ128::CFP and mSc::IFD-1. Neurons were imaged at adult-age, with animals rescued from microscope slides and placed back on plates. Three days later, the same neuron was imaged. Two more days later (+5 days) the neuron was imaged again. Strain is expressing *igIs1*[P*_mec-7_* YFP, P*_mec-3_* Htt57Q128::CFP, lin-15(+)]; *bzSi34* [P*_mec-7_* mScarlet::IFD-1]. Of nine multi-day observations of mSc and CFP interactions, about half the time (4/9 observations) HttQ128 globularly expanded at the mSc::IFD-1-aggresome-like site. **b** Quantification of HttQ128 collection at the aggresome-like site over time. We imaged touch neurons that co-expressed interacting HttQ128::CFP and mSc::IFD-1 at time 1 (nine animals distributed over the X-axis). We then imaged the same neurons again, at least 2 days later (time 2). Following the same aggresome-like site over a period of at least 2 days, we see HttQ128::CFP can exhibit a dynamic collection pattern. HttQ128 can either globularly expand at the aggresome-like site (as measured by intensity, AU) in 4/9 neurons (and as depicted in Panel a), or decrease in intensity at the aggresome site, observed in 5/9 neurons. **c** Quantification of mSc::IFD-1 collection at the HttQ128-filled aggresome-like organelles. In the same experiment as in **b**, we scored the same aggresome-like site over a period of at least two days, to document that mSc::IFD-1 typically displays a relative reduction in collection over time, as measured by maximum intensity (arbitrary units) at the aggresome-like site (8/9 neurons displayed reduced intensity at the aggresome-like site). Interestingly, aggresome dynamics in the absence of aggresome-targeted protein HttQ128 appear different (only GFP::IFD-1 observed over time; see Supplementary Fig. 1b which shows GFP::IFD-1 aggresome-like growth over time in the absence of aggresome-targeting-protein, HttQ128). Our data suggest alternative dynamics of the IF-aggresome-like organelle in the presence of an elevated aggresome-targeted-aggregate like HttQ128. Note that the X-axis is plotted to show trend of growth between timepoint 1 and timepoint 2 and aggresome-like sites cannot be compared between panels **b** and **c**.

**Supplementary Fig. 5. Over-expressed mCherry is predominantly housed in LMP-1-bound lysosomal-organelles, which are distinct from the IF-compartment. a** mCherry is mostly in LMP-1-bound structures. A representative adult ALM neuron co-expressing mCherry and the LMP-1::GFP lysosome marker (white solid line outlines the soma cell body and the dotted line outlines the nucleus). Representative of N > 250. LMP-1::GFP signal is enriched in the lysosome membrane forming a GFP ring (pseudo-colored blue) around the mCherry collection. mCherry degradation appears minimal within the LMP-1::GFP compartment as signal persists. GFP channel is pseudo-colored to blue to emphasize reporter difference between panels **a** and **b**. Strain is expresssing bzIs166[P_mec-4_mCherry]; bzIs168[P_mec-7_LMP-1::GFP]. Scale bar = 2 µm. **b** IFD-positive organelles are often localized next to, but not overlapping with, a subset of mCherry concentrations when tagged IFD is expressed from a single copy transgene. Representative Ad2 touch neuron, representative of N > 50. Strain is bzIs166[P_mec-4_mCherry]; *bzSi3*[P_mec-7_GFP::IFD-1]; scale bar = 2 µm.

**Supplementary Fig. 6. General health, development, lifespan, and stress response measures are largely preserved in the *ifd* null mutants. a** Body length in µM. Synchronized Ad2 animals. N = 31, 33, 32 animals for control, *ifd-1(ok2404)*, and *ifd-2(bz187)* respectively. P = NS, 3 trials, one-way ANOVA with Dunnett’s post-test. **b** Body width as measured from 10x images taken from dissection microscope. Ad2 animals. N = 21 adults for all strains, P = NS, 3 trials, one-way ANOVA with Dunnett’s post-test. **c** Progeny number per adult. N = 9 adults for all strains, P = NS, 3 trials, one-way ANOVA with Dunnett’s post-test. **d-e** Hours to develop from egg to young adult stage. For N collection, we performed a 1 hour egg lay with 50 gravid animals per strain, then removed the parents and let the animals grow. N > 800 animals followed to adulthood, percentage of the population to develop is graphed. 3 trials, unpaired two-tailed t-test where mean values of 3 trials were considered using GraphPad Prism.^10^ Error bars are SEM. **d** Development for *ifd-1(ok2404)*. P = 0.72,. **e** Development for *ifd-2(bz187)*. P = 0.38. **f-g** Survival curve. Graphed as days until death (percentage of surviving animals as a function of days of adulthood) N = 167 and 180 animals followed for the control and *ifd-1* respectively. *ifd-1* P = 0.9442; N = 165 and 180 animals followed for the control and *ifd-2* respectively, *ifd-2*  P = 0.1152. 3 trials. Error bars are SEM. We performed statistical analysis using the survival function and the Gehan-Breslow-Wilcoxon Test of GraphPad Prism.^10^ **f** *ifd-1(ok2404); bzIs166*[P*_mec-_*4mCherry]. **g** *ifd-2(bz187); bzIs166*[P*_mec-_*4mCherry]. **h** Percent survival after 24 hours of exposure to 450 mM NaCl concentrated plates; strains *ifd-1(ok2404);bzIs166*[P*_mec-_*4mCherry] and *ifd-2(bz187); bzIs166*[P*_mec-_*4mCherry]. The viability of animals after overnight exposure to NaCl was scored. 5 replicates with 60 animals per condition, N = 300 animals per condition, the animals were followed until they died; graphed is the percentage of surviving animals. *ifd-1* P = 0.60, *ifd-2* P = 0.92, error bars are SEM; one-way ANOVA with Dunnett’s post-test using GraphPad Prism^10^. **i** Hours on paraquat vs. survival; strains *ifd-1(ok2404);bzIs166*[P*_mec-_*4mCherry] and *ifd-2(bz187); bzIs166*[P*_mec-_*4mCherry]. N = 180 animals per strain*, ifd-1* ***P = 0.0004, *ifd-2* P = 0.076, 3 trials, error bars are SEM. We performed statistical analysis using the survival function and the Gehan-Breslow-Wilcoxon Test of GraphPad Prism.^10^ **j-k** GST-4::GFP is a common reporter of cytosolic stress. Maximum GFP intensity per animal reported in arbitrary units. 3 trials, two -tailed t-test, error bars are SEM. **j** GST-4::GFP expression is NS (P = 0.16) in *ifd-1* mutants. Strains are *dvIs19[(*pAF15)P*gst-4*GFP::NLS] and *dvIs19*[(pAF15) P*gst-4*GFP::NLS]; *ifd-1(ok2404*); N = 31 and 30 animals respectively. **k** GST-4::GFP expression is reduced in *ifd-2* animals (P = 0.0256). Strains are *dvIs19*[(pAF15) P*gst-4*GFP::NLS] and *dvIs19*[(pAF15) P*gst-4GFP*::NLS]; *ifd-2(bz187);* N= 31 and 32 animals respectively.

==

**Supplementary Fig. 7 Touch neuron integrity and function are largely preserved in the *ifd* null mutants.** **a** Mechanosensory touch function on Ad1, Ad5, and Ad10 for *ifd-1(ok2404);bzIs166*[P*_mec-_*4mCherry] and *ifd-2(bz187);bzIs166*[P*_mec-_*4mCherry]. Graphed is percentage response to fine touch. P = NS, one-way ANOVA with Dunnett’s post-test, Ad1 - 3 trials, N = 63, 63, and 62 animals per strain, Ad5 - 1 trial N = 29, 31, and 30 animals per strain, Ad10 - 1 trial, N = 30 animals per strain. Error bars are SEM. **b** ALM morphology of strains *ifd-1(ok2404);bzIs166*[P*_mec-_*4mCherry] and *ifd-2(bz187);bzIs166*[P*_mec-_*4mCherry]. We scored axonal beading and soma outgrowth percentage on Ad2. Beading and outgrowths are associated with age, stress, and neurodegeneration^11^. NS, one-way ANOVA N = 38, 36, and 33 ALMs for control, *ifd-1,* and *ifd-2* mutant strains respectively. 3 trials, error bars are SEM. **c** *ifd-2* mutants display normal number of mCherry collections per ALM. Animals expressing *bzIs166*[P*_mec-4_* mCherry] with the *ifd-1* or *ifd-2* mutation have the same number of mCherry aggregates as wildtype – 4.67, 4.56, and 4.31 mean mCherry collections per cell respectively for control, *ifd-1*, and *ifd-2*. N = 42, 39, 41 ALMs respectively, one-way ANOVA with Dunnett’s post-test, 3 trials, error bars are SEM, P = 0.98 for *ifd-1,* P = 0.79 for *ifd-2.* **d** *ifd-2* mutants display normal size of mCherry collections per ALM. The mean diameter of the mCherry puncta in WT animals expressing *bzIs166*[P*_mec-4_* mCherry] is 8.34 arbitrary units; there is a NS change compared to the *ifd-1* mutant (8.57 arbitrary unit mean diameter) or *ifd-2* mutant (8.88 arbitrary unit mean diameter). N = 42, 39, 41 ALM respectively, one-way ANOVA with Dunnett’s post-test, 3 trials, error bars are SEM, P = 0.69 for *ifd-1,* P = 0.15 for *ifd-2.*

**Supplementary Fig. 8. *ifd-1* and *ifd-2* deletions decrease ALMR exophers as reported by a highly expressed cytosolic GFP reporter. a** Representative Ad2 exopher produced from touch neurons in *uIs31*[P*_mec-17_*GFP]. **b** We scored ALMR exopher production on Ad2 in the *ifd-1(ok2404)* deletion mutant and the *ifd-2(bz187)* deletion mutant, each of which also harbored *uIs31*[P*_mec-17_*GFP] integrated transgene. N = 419, 392, and 33l ALMR for control and each mutant respectively, 6 trials, error bars are SEM, Cochran-Mantel-Haenszel test; *ifd-1*(P = 0.0000012); *ifd-2* (P = 0.000018). Data support that *ifd* disruptions modulate exopher levels independently of the fluorescent reporter used for exopher detection.

**Supplementary Fig. 9. RNAi knockdown of intermediate filament genes can decrease exopher production.** Because some exopher production is evident when *ifd-1* and *ifd-2* are both absent (Fig. 4), we infer that a redundant activity or a parallel pathway must also contribute to exopher formation. **a** We performed knockdown of each of 9 *C. elegans* intermediate filament genes, via feeding RNAi for 2 generations, ALMR exophers assayed on Ad2, using strain expressing *bzIs166* [P*_mec_-4*mCherry1] II; *sid-1(qt9)* V; *sqIs71* [P*rgef-1*GFP; P*rgef-1sid-1*;pBS]. RNAi should be effective only in all neurons in this strain. N = 250 ALMR/clone, 5 trials, error bars are SEM; Cochran-Mantel-Haenszel test (CMH); *ifd-1* P = 0.13; *ifa-2* ***P = 0.00019; *ifa-3* *P = 0.020; *ifb-1* **P = 0.0036; *ifb-2* *P = 0.045; *ifc-1* *P = 0.017; *ifc-2* *P = 0.046; *ifd-1* **P = 0.0020; *ifd-2* *P = 0.011. **b** IF RNAi knockdown in the *ifd-1(Δ); ifd-2(Δ)* double mutant background can exacerbate decreases in exopher percentage to less than 5%. RNAi in strain *ifd-1(ok2404); ifd-2(bz187);* *bzIs166*[P*_mec-_*4mCherry], *uIs71*[(pCFJ90) P_myo_*_-2_*mCherry + P*_mec-18_*SID-1] exposed from L4-Ad2; ALMR exophers assayed Ad2. Because in this strain, all cells including touch neurons, but no other neurons but should be targeted; RNAi disruptions might not act touch neuron autonomously due to RNAi susceptibility of other cells. RNAi knockdowns were 200 ALMR/clone, 4 trials. Subsets of experimental RNAi clones were assayed on the same day and therefore used the same empty vector control; each clone is graphed separately with day-matched EV controls. Since very low baseline exopher levels are present for the *ifd-1(Δ); ifd-2(Δ)* starting strain, any role implied by these RNAi data requires confirmation by independent genetic perturbation and cell autonomy analyses. Cochran-Mantel-Haenszel test; error bars are SEM; *ifa-1* P = 0.17, 3 trials, N = 150 ALMR per clone; *ifa-2* *P= 0.024, 4 trials, N = 200 ALMR per clone; *ifa-3* ***P = 0.00035, 3 trials, N = 150 ALMR per clone; *ifb-1* P = 0.17, 3 trials, N = 150 ALMR per clone; *ifb-2* ***P = 0.00011, 4 trials, N = 200 ALMR per clone; *ifp-1* P = 0.053, 5 trials, N = 250 ALMR per clone; *ifc-1* P = 0.22, 4 trials, N = 200 ALMR per clone; *ifc-2* P = 0.00031, 4 trials, N = 200 ALMR per clone; *ifd-1* P = 0.89, 4 trials, N = 200 ALMR per clone; *ifd-2* P = 0.084, 4 trials, N = 200 ALMR per clone.

**Supplementary Fig. 10. Intestine-specific *ifd-2* expression is not effective for significant rescue of *ifd-2(****Δ****)* defects in exopher production.** Statistics with Cochran-Mantel-Haenszel test, error bars SEM. *ifd-2* is expressed in intestine and can influence intestinal morphology^10^, raising the question as to whether the intestine might signal non-autonomously to impact exopher production. We therefore expressed *ifd-2* from the *vha-6* intestine-specific promoter in the *ifd-2(Δ)*; mCherry background and scored exopher levels. We compared ALMR exophers at Ad2 in *bzIs166*[P*_mec-4_*mCherry] compared to *bzIs166*[P*_mec-4_*mCherry]; *ifd-2(bz187)* (P = 0.0054); *bzIs166*[P*_mec-4_*mCherry] compared to *bzIs166*[P*_mec-4_*mCherry]; *ifd-2(bz187); bzSi45*[P*_vha-6_*mSc::IFD-2] (P = 0.29). *bzIs166*[P*_mec-4_*mCherry]; *ifd-2(bz187)* compared to *bzIs166*[P*_mec-4_*mCherry]; *ifd-2(bz187); bzSi45*[P*_vha-6_*mSc::IFD-2] is P = 0.07, N = 219, 197, and 208 ALMR for control, mutant, and mutant with transgene, respectively. 5 trials. Comparing *ifd-2(bz187)* to *ifd-2(bz187);*P*_vha-6_*mNeonGreen::IFD-2, we find that intestinal expression of mNeonGreen::IFD-2 does not rescue the neuronal exopher phenotype of *ifd-2(bz187),* consistent with a predominant role for *ifd-2* in exophergenesis in the neuron (Fig. 4e-g). Still, the trend toward partial rescue leaves open the possibility of some intestinal contribution. Note: We could not detect expression from native *ifd* promoters or native *ifd* transgenes in touch neurons, a reporter outcome we often observe for native promoter-single copy reporters including touch neuron channel *mec-4*, dynein heavy chain *dhc-1*, and *rab-11,* a GTPase required for recycling endosome function, for examples^12,13^**.**

**Supplementary Fig. 11. Transgenic IF proteins are neither dominant negative for exopher production nor exopher-elevating on their own.** Statistics with Cochran-Mantel-Haenszel test, error bars SEM. **a** Single copy *bzIs3*[P*_mec-7_*GFP::IFD-1] does not affect Ad2 ALMR exopher levels. 10 trials, P = 0.92, strains are *bzIs166*[P*_mec-4_*mCherry] and *bzIs166*[P*_mec-4_*mCherry];*bzSi3*[P*_mec-7_*GFP::IFD-1], N = 918 and 573 ALMR respectively.  **b** Single copy *bzIs37*[P*_mec-7_*mNG::IFD-2] does not affect Ad2 ALMR exopher percentage. 3 trials, P = 0.75, strains are *bzIs166*[P*_mec-4_*mCherry] and *bzIs166*[P*_mec-4_*mCherry];*bzSi37*[P*_mec-7_*mNeonGreen::IFD-2], N = 101 and 103 ALMR respectively. **c** Over-expressed (OE) GFP::IFD-1 array does not affect Ad2 ALMR exopher percentage. Strains are *bzIs166*[P*_mec-4_*mCherry] and *bzIs166*[P*_mec-4_*mCherry];*bzEx270*[P*_mec-7_*GFP::IFD-1]. N = 267 and 209 ALMR respectively, P = 0.0904, 7 trials.  **d** Over-expressed (OE) RFP::IFD-2 array does not affect Ad2 ALMR exopher percentage. Strains are *bzIs166*[P*_mec-4_*mCherry] and *bzIs166*[P*_mec-4_*mCherry];*bzEx253*[P*_mec-7_*RFP::IFD-2], N = 470 and 426 ALMR respectively; 11 trials, P = 0.99.  **e** An added single copy GFP::IFD-2 transgene expressed from the native *ifd-2* promoter does not change exopher levels. We expressed GFP::IFD-2 from the native *ifd-2* promoter; mCherry background, and scored for exopher levels. We compared ALMR exophers at Ad2 in *bzIs166*[P*_mec-4_*mCherry] to *bzSi76*[P*_ifd-2_*GFP::IFD-2] and found no significant difference, N = 317 and 318 ALMR respectively; P = 0.15, 6 trials. Our data suggest that the *ifd-*dependent mechanisms influencing exopher levels are unlikely to be strictly dependent on stoichiometric interactions with concentration-limited partner proteins or be anchored in simple enhanced *ifd* expression levels. Lack of over-expression effects are consistent with our observation that size of aggresome compartment per se does not correlate with generally high exopher levels nor does it predict extrusion (Supplementary Fig. 11, Supplementary Fig. 13).

**Supplementary Fig. 12. Touch neuron-specific tagged high-copy number IFD transgenes can rescue the IF exopher deficit.** Statistics with Cochran-Mantel-Haenszel test, error bars SEM. **a** We scored ALMR exophers at Ad2 in *bzIs166*[P*_mec-4_*mCherry] compared to *bzIs166*[P*_mec-4_*mCherry]; *ifd-1(ok2404),* (*P = 0.018). *bzIs166*[P*_mec-4_*mCherry] compared to *bzIs166*[P*_mec-4_*mCherry]; *ifd-1(ok2404); bzEx253*[P*_mec-7_*GFP::IFD-2 OE] is NS (P = 0.0642). N = 470, 470, and 435 ALMR respectively*;* 11 trials. **b** We scored ALMR exophers at Ad2 in *bzIs166*[P*_mec-4_*mCherry] compared to *bzIs166*[P*_mec-4_*mCherry]; *ifd-2(bz187)* (*P = 0.0057). *bzIs166*[P*_mec-4_*mCherry] compared to *bzIs166*[P*_mec-4_*mCherry]; *ifd-2(bz187); bzEx253*[P*_mec-7_*RFP::IFD-2 OE] is NS (P = 0.623), N = 241, 294, 257 ALMR respectively, 7 trials. Cross complementation might reflect a need for a particular level for function that cannot be reached from normal gene dosages of *ifd-1* or *ifd-2*.

**Supplementary Fig. 13. The size of the GFP::IFD-1 concentration does not correlate with exopher rate. a** GFP::IFD-1-aggresome-like organelles can be cleared from the soma, but the size of aggresome-like organelles that frequently remain in the soma does not correlate with an exopher event. We imaged 95 Ad3 ALMs from strain expressing *bzIs166*[P*_mec-4_*mCherry];*bzSi3*[P*_mec-7_*GFP::IFD-1]. 51/95 (54%) neurons displayed an exopher event (exopher population) while 44/95 (46%) did not (‘no exopher’ population). We measured the diameter (in arbitrary units) of the aggresome-like organelle in the soma in both groups. We found that the ‘exopher+ population’ included several cases in which there was no remaining GFP::IFD-1 aggresome-like organelle in the soma (7/44, or ~16%) (consistent with the idea that the neuron ejects GFP::IFD-1 aggresome-like organelles discussed in Fig. 5b, showing ~15% GFP::IFD-1-aggresome-like organelle ejection rate). There was one case in which there was no detectable GFP::IFD-1 aggresome-like organelle in the ‘no-exopher’ population. Not considering cases in which there was not a GFP::IFD-1 aggresome-like organelle to measure, we measured no significance in the size of the soma located aggresome-like organelle between the population of animals that made an exopher versus the population that did not make an exopher. P = 0.219 excluding datapoints with 0 aggresome, two-tailed t-test, error bars are SEM. **b** Early (L4) aggresome-like organelle size does not predict Ad1-Ad3 exophergenesis. To ask if early aggresome-like organelle size could indicate likelihood of later exophergenesis on Ad2 or 3, we measured the L4 aggresome-like organelle size and number per cell, and later scored for exopher production. We imaged L4 neurons and successfully recovered 154 animals post-imaging. After 2-3 days on NGM-plates, we scored for exophers (Ad2 and Ad3). Out of 154 animals, 24 had an ALM exopher evident on Ad2/Ad3. To analyze, we binned the animals into two populations: one population that did not make an exopher (‘no-exopher’) and a population that that did make an exopher (‘exopher’). We found no difference in early (L4) aggresome-like organelle number per cell between the two populations. P = 0.67, two-tailed t-test, N successfully scored on Ad2 = 50, 31, 18, 24, and 26 in 5 biologic trials, error bars are SEM. **c** Continuing analysis from **b**, we measured GFP::IFD-1 diameter (arbitrary units) in the L4 ALMs in both populations and found NS, P = 0.47 two-tailed t-test, error bars are SEM.

**Supplementary Fig. 14. Adaptor protein impact on IFD compartments and exopher production: homologs of mammalian aggregate-adapter complex members FTT-2 and HSP-1 have roles in exopher production. a** We scored ALMR exophers on Ad2 in *bzIs101*[P*_mec-4_*mCherry] compared to *bzIs166*[P_mec-4_mCherry]; *sqst-1(ok2892)*; N = 367 and 349 ALMR respectively, NS, P = 0.78, 9 trials, Cochran-Mantel-Haenszel (CMH) test. Boxes represent the range, minimum (9.3%, 5.9%), maximum (26%, 30%), and mean (17%, 24%) for control and mutant, respectively. **b-f** Error bars are SEM. **b** Strains are *bzIs166*[P*_mec-4_*mCherry]; *hda-6(ok3203),* *bzSi3*[P*_mec-7_*GFP::IFD-1] and *bzIs166*[P*_mec-4_*mCherry]; *bzSi3*[P*_mec-7_*GFP::IFD-1]; ****P = 0.0001, N > 150, 8 trials. Two-tailed t-test. **c** There is a significant reduction of percentage of ALMR exopher production on Ad1 in the genomic deletion *ftt-2(n4426*) strain expressing *bzIs166*[P*_mec-4_* mCherry]; *bzSi3*[P*_mec-7_* GFP::IFD-1], N = 276 and 354 ALMR respectively; **P = 0.00908, 8 trials, CMH test. Measures on Ad1 avoid animal-bagging consequences. **d** We knocked down *ftt-2* in a touch neuron-sensitized RNAi strain expressing *bzIs169*[P*_mec-18_*SID-1 P_sng-1_YFP] (other cells and touch neurons targeted in this background; but no other neurons) from L4 - Ad2; N = 86 and 110 ALMR for empty vector and knockdown respectively, **P = 0.0032, 3 trials, CMH test. See note in Methods regarding fertility. **e** We targeted *ftt-2* knockdown (2 generation RNAi treatment) in a pan-neuronal-specific RNAi strain expressing *sqIs71*[P*_rgef-1_*GFP; P*_rgef-1_*SID-1;pBS]; *sid-1(qt9)*V; *bzIs101*[P*_mec-4_*mCherry] (only neurons subject to *ftt-2* knockdown) to observe reduced Ad2 ALMR exophergenesis, **P = 0.0025, 5 trials, N = 250 ALMR for each condition, CMH test. **f** We scored ALMR exopher production on Ad2 in control animals expressing *bzIs166*[P*_mec-4_*mCherry] and *bzIs166*[P*_mec-4_*mCherry]; *bzSi47*[P*_mec-7_*FTT-2::mSc], N = 101 and 97 ALMR, respectively. Note that the wild type *ftt-2(+)* allele is present and that use of the strong *mec-7* promoter for TN specific expression of the added *ftt-2* copy might elevate expression. Single-copy integrated P*_mec-7_*FTT-2::mSc increases ALMR Ad2 exophergenesis, CMH test; *P = 0.035, 3 trials. **g** 14-3-3 proteins bind directly to dynein components. 14-3-3 binds BAG3 bound Hsc70/(HSP-1). Hsc70/HSP-1 binds ubiquitinylated aggregates such that the complex moves along the microtubule to deliver aggregates to the aggresome compartment. **h** *hsp-1* RNAi from L4 to Ad2 in a touch neuronal sensitized RNAi strain harboring *bzIs169* [P*_mec-18_sid-1* P_s_*_ng-1_*YFP]; *bzIs101* [P*_mec-4_*mCherry] (targeting all cells + touch neurons) reduces ALM exophergenesis; ***P = 0.0000050, N = 138 and 142 Ad2 ALMR for empty vector and knockdown respectively, 4 trials, CMH, error bars are SEM.

**Supplementary Fig. 15. Human intermediate filament Neurofilament Light Chain (hNFL) can adopt multiple localization patterns in *C. elegans* touch neurons, but prominently localizes to 1 or 2 juxtanuclear inclusions. a-d)** Human hNFL can adopt multiple localization patterns when expressed in the *C. elegans* touch neurons **– a** filamentous/cytoplasmic (Strain is *bzEx311*[P*_mec-7_*mNG::hNFL];*bzIs166*[P*_mec-4_*mCherry]; *ifd-2(bz187)*)*.* **b** axonal (and punctate featured). **c** nuclear (and punctate featured). And **d** most common: juxtanuclear and punctate. **b-e** Strain is *bzEx269*[P*_mec-7_*mNG::hNFL];*bzSi34*[P*_mec-7_*mSc::IFD-1]. **e** Top - filamentous/ cytosolic localization. Bottom – typical punctate/collected localization. **f-k** Human mNG::hNFL forms juxtanuclear inclusions in either single copy or high copy array lines. **f-h** quantification of integrated single copy nNFL strain expressing *bzSi48*[P*_mec-7_* mNG::hNFL]; *bzIs166*[P*_mec-4_*mCherry]. **i-k** Quantification of high copy array. Strain expressing *bzEx269*[P*_mec-7_*mNG::hNFL];*bzSi34*[P*_mec-7_*mSc::IFD-1]. Collected or cytosolic localization phenotype quantified in **f** and **i**. Juxtanuclear location quantified in **g** and **j**. (**f-g, i-j:** 3 trials graphed, N > 30 ALM total.) Number of collected punctae per ALM quantified in **h** and **k** (N = 40 and 33 ALMR respectively). Error bars SEM.

**Supplementary Fig. 16. Single copy transgenic hNFL tagged protein is not dominant negative for exopher production or exopher-elevating on its own.** Single copy mNG::hNFL does not significantly affect Ad2 ALMR exopher percentage. Strains are *bzIs166*[P*_mec-4_*mCherry] and *bzIs166*[P*_mec-4_*mCherry];*bzSi48*[P*_mec-7_*mNG::hNFL]; N = 95 and 117 ALMR respectively; 3 trials, Cochran-Mantel-Haenszel test, P = 0.44, error bars SEM.

**Supplementary Fig. 17. Summary model of *C. elegans* aggresome-like organelle formation, ejection, and the requirements of efficient exophergenesis. A** *C. elegans* mechanosensory touch neurons typically contain 1-3 aggresome-like intermediate filament-decorated collections that can contain ubiquitin (UBQ-2) and aggregated proteins (HttQ74 and HttQ128). Adapter proteins (FTT-2/14-3-3), from the 14-3-3/BAG3/Hsc70 complex) shuttle ubiquitin tagged aggregated proteins facilitated by dynein transport on the microtubule track for juxtanuclear collection. Furthermore FTT-2(14-3-3) and HSP-1(Hsc70) (as well as ubiquitin and HttPolyQ aggregates, and SQST-1, a potential alternative aggresome-adaptor) can colocalize with the IF-aggresome-like site. One large IF-aggresome-like organelle is depicted to emphasize detail, and a smaller IF-aggresome-like collection is shown on the other side of the nucleus. Note that the IF-aggresome-like organelle can contain aggregate compactions that are not encased or fully colocalized with IF-protein. In situations of high proteostress, *C. elegans* touch neurons can facilitate aggregate ejection via exophergenesis (outcome 2 and 3). The IF-organelle and its contents can be ejected in the exopher (~15-50% of exopher events, Fig. 5) (option 2), or can remain in the soma as other contents can be expelled (option 3). **b** Without intermediate filament proteins IFD-1 and IFD-2 there is reduced exophergenesis. IFD-1 and IFD-2 act cell autonomously, within the neuron, in the exopher pathway. Aggresome adaptor proteins FTT-2(14-3-3), and HSP-1(Hsc70) of the 14-3-3/BAG3/Hsc70 adapter complex are also required for efficient exophergenesis, linking proteins in aggresome biology with cellular extrusion functions. FTT-2, which is hypothesized to work with HSP-1 in the IF-aggresome formation pathway, is required for efficient IFD-collection at the aggresome site. With *ftt-2* knockout or knockdown, there is reduced IFD-collection at the juxta nuclear aggresome-like site.

**Supplementary Fig. 18. IFD-1-puncta form without *ifd-2*. a IFD-1=puncta** diameter, in arbitrary units, in Ad2 ALM neurons with and without *ifd-2* in strains expressing *bzIs166*[P*_mec-4_* mCherry]; *bzSi3*[P*_mec-7_* GFP::IFD-1]; N = 55 and 58 puncta respectively; P = NS, 3 trials, two-tailed t-test, error bars are SEM. **b** IFD-1-puncta form juxtanuclearly with or without *ifd-2.* Percentage of juxtanuclear IFD-1-puncta per ALM does not significantly differ in strains expressing *bzIs166*[P*_mec-4_* mCherry]; *bzSi3*[P*_mec-7_* GFP::IFD-1], with or without likely null allele *ifd-2(bz187)*; N = 40 and 37 ALMs respectively; P = NS, 3 trials, two-tailed t-test, error bars SEM. Juxtanuclear location is assessed in a binary manner. **c** IFD-1-puncta number per ALM with or without *ifd-2(bz187*) in strains expressing *bzIs166*[P*_mec-4_* mCherry]; *bzSi3*[P*_mec-7_* GFP::IFD-1] remains the same; N = 41 and 39 ALMs respectively; P = NS, 3 trials, two-tailed t-test, error bars are SEM.

**Supplementary Data 1.** Table including information on strains, alleles, and RNAi plasmids.

**Supplementary References**

1 Lehotzky, A. *et al.* Dynamic targeting of microtubules by TPPP/p25 affects cell survival. *J Cell Sci* **117**, 6249-6259, doi:10.1242/jcs.01550 (2004).

2 Kopito, R. R. Aggresomes, inclusion bodies and protein aggregation. *Trends Cell Biol* **10**, 524-530, doi:10.1016/s0962-8924(00)01852-3 (2000).

3 Johnston, J. A., Ward, C. L. & Kopito, R. R. Aggresomes: a cellular response to misfolded proteins. *The Journal of cell biology* **143**, 1883-1898 (1998).

4 García-Mata, R., Bebök, Z., Sorscher, E. J. & Sztul, E. S. Characterization and dynamics of aggresome formation by a cytosolic GFP-chimera. *The Journal of cell biology* **146**, 1239-1254, doi:10.1083/jcb.146.6.1239 (1999).

5 Kolodziejska, K. E., Burns, A. R., Moore, R. H., Stenoien, D. L. & Eissa, N. T. Regulation of inducible nitric oxide synthase by aggresome formation. *Proc Natl Acad Sci U S A* **102**, 4854-4859, doi:10.1073/pnas.0500485102 (2005).

6 Saliba, R. S., Munro, P. M., Luthert, P. J. & Cheetham, M. E. The cellular fate of mutant rhodopsin: quality control, degradation and aggresome formation. *J Cell Sci* **115**, 2907-2918, doi:10.1242/jcs.115.14.2907 (2002).

7 Fortun, J., Dunn, W. A., Jr., Joy, S., Li, J. & Notterpek, L. Emerging role for autophagy in the removal of aggresomes in Schwann cells. *J Neurosci* **23**, 10672-10680, doi:10.1523/jneurosci.23-33-10672.2003 (2003).

8 Viswanathan, J. *et al.* Alzheimer's disease-associated ubiquilin-1 regulates presenilin-1 accumulation and aggresome formation. *Traffic* **12**, 330-348, doi:10.1111/j.1600-0854.2010.01149.x (2011).

9 Gautreau, A. *et al.* Isolation and characterization of an aggresome determinant in the NF2 tumor suppressor. *J Biol Chem* **278**, 6235-6242, doi:10.1074/jbc.M210639200 (2003).

10 Geisler, F. *et al.* Intestinal intermediate filament polypeptides in C. elegans: Common and isotype-specific contributions to intestinal ultrastructure and function. *Scientific Reports* **10**, 3142, doi:10.1038/s41598-020-59791-w (2020).

11 Toth, M. L. *et al.* Neurite sprouting and synapse deterioration in the aging Caenorhabditis elegans nervous system. *J Neurosci* **32**, 8778-8790, doi:10.1523/jneurosci.1494-11.2012 (2012).

12 Lai, C. C., Hong, K., Kinnell, M., Chalfie, M. & Driscoll, M. Sequence and transmembrane topology of MEC-4, an ion channel subunit required for mechanotransduction in Caenorhabditis elegans. *J Cell Biol* **133**, 1071-1081, doi:10.1083/jcb.133.5.1071 (1996).

13 Zhang, L., Ward, J. D., Cheng, Z. & Dernburg, A. F. The auxin-inducible degradation (AID) system enables versatile conditional protein depletion in C. elegans. *Development (Cambridge, England)* **142**, 4374-4384, doi:10.1242/dev.129635 (2015).
